# Reply to Bouva et al. Comment on “Dijkstra et al. A False-Negative Newborn Screen for Tyrosinemia Type 1—Need for Re-Evaluation of Newborn Screening with Succinylacetone. *Int. J. Neonatal Screen.* 2023, *9*, 66”

**DOI:** 10.3390/ijns10040066

**Published:** 2024-09-24

**Authors:** Allysa M. Dijkstra, Kimber Evers-van Vliet, M. Rebecca Heiner-Fokkema, Frank A. J. A. Bodewes, Dennis K. Bos, József Zsiros, Koen J. van Aerde, Klaas Koop, Francjan J. van Spronsen, Charlotte M. A. Lubout

**Affiliations:** 1 Section of Metabolic Diseases, Beatrix Children’s Hospital, University Medical Center Groningen, University of Groningen, 9700 RB Groningen, The Netherlandsf.j.van.spronsen@umcg.nl (F.J.v.S.); 2 Laboratory of Metabolic Diseases, Department of Laboratory Medicine, University Medical Center Groningen, University of Groningen, 9700 RB Groningen, The Netherlands; m.r.heiner@umcg.nl; 3 Section of Pediatric Gastroenterology and Hepatology, Beatrix Children’s Hospital, University Medical Center Groningen, University of Groningen, 9700 RB Groningen, The Netherlands; 4 Department of Genetics, University Medical Center Groningen, University of Groningen, 9700 RB Groningen, The Netherlands; 5 Princess Máxima Center for Pediatric Oncology, 3584 CX Utrecht, The Netherlands; 6 Department of Pediatric Infectious Disease and Immunology, Amalia’s Children Hospital, Radboud University Medical Center, 6525 GA Nijmegen, The Netherlands; 7 Section Metabolic Diseases, Department of Pediatrics, University Medical Center Utrecht, 3584 CX Utrecht, The Netherlands

We thank the authors for their comments [1]. We are happy that they agree with the content of the report [2] and underline the importance of having optimal cut-off values of succinylacetone in order to—on the one hand—not miss patients like the one presented in this report and, on the other hand, to exclude false positive results, for example, due to a MAAI deficiency. As stated in the original article (Van Vliet et al. 2023, [3]), this might mean that the succinylacetone cut-off in the newborn screening needs to be lowered, while adding additional biomarkers that can be used to distinguish children with mild Tyrosinemia type I who need treatment from those with MAAI deficiency, who do not need treatment at all.

Regarding this aim, the results from the mutual project on the improvement of newborn screening for Tyrosinemia type I, as mentioned in the comment, are needed. 

We further call attention to other institutes who have children with a (suspected) MAAI deficiency to send us dried blood spots and urine to increase our knowledge on MAAI deficiency.
